# The Prognosis Role of AST/ALT (De Ritis) Ratio in Patients with Adult Secondary Hemophagocytic Lymphohistiocytosis

**DOI:** 10.1155/2020/5719751

**Published:** 2020-12-05

**Authors:** Guangli Yin, Changfeng Man, Shengen Liao, Hongxia Qiu

**Affiliations:** ^1^Department of Hematology, The First Affiliated Hospital of Nanjing Medical University, Jiangsu Province Hospital, 300 Guangzhou Road, Nanjing 210029, China; ^2^Department of Cardiology, The First Affiliated Hospital of Nanjing Medical University, 300 Guangzhou Road, Nanjing 210029, China; ^3^Department of Geriatric Hematology, The First Affiliated Hospital of Nanjing Medical University, Jiangsu Province Hospital, 300 Guangzhou Road, Nanjing 210029, China

## Abstract

**Purpose:**

Secondary hemophagocytic lymphohistiocytosis (sHLH) accompanied by liver involvement, characterized by hepatomegaly and increased liver enzymes, is usually associated with elevated mortality. However, the magnitude of these associations remains unknown. Our objective was to assess the associations of the aspartate transaminase/alanine transaminase (AST/ALT, De Ritis) ratio with overall survival among adult patients with sHLH.

**Methods:**

A retrospective analysis was performed on 289 patients aged 18–86 years with complete serum transaminase data at diagnosis of sHLH. Multivariate Cox regression analyses and restricted cubic splines were conducted to address the association between the De Ritis ratio and the risk of mortality.

**Results:**

The median De Ritis ratio for the entire study population was 1.34 (IQR: 0.84-2.29). After a median follow-up time of 60 (range 17-227.5) days, 205 deaths occurred. After fully adjusting for hepatomegaly, albumin, fibrinogen, EBV, ferritin, etiologies, and treatment strategies, the adjusted hazard ratios (HRs) with corresponding confidence intervals (CIs) of mortality for the 2 st tertile and 3 st tertile were 1.2 (0.8-1.7) and 1.6 (1.1-2.2), respectively (*P* < 0.01 for trends). Restricted cubic spline confirmed a linear association between the log_2_-transformed De Ritis ratio and the risk of mortality. Moreover, this trend persisted in subgroups with MHLH, hyperferrinaemia, sCD25 ≤ 20,000 ng/L, patients without EBV infection, and those received treatment.

**Conclusions:**

The De Ritis ratio is a strong and independent predictor for overall survival in patients with sHLH. As a readily available biomarker in routine clinical practice, it is used to identify patients with sHLH with inferior overall survival.

## 1. Introduction

Adult secondary hemophagocytic lymphohistiocytosis (sHLH) is an immune-mediated life-threatening disease characterized by aberrant activation and proliferation of polyclonal T lymphocytes and macrophages, leading to a cytokine storm, haemophagocytosis, and multiorgan infiltration and dysfunction [1]. Owing to a lack of awareness of the severity of the disease and prognosis, a delay in prompt treatment contributes to the high mortality rates. Therefore, it is becoming more important to identify prognostic indicators among patients with sHLH. For clinical practice ascertainment, a potential prognostic indicator would generally have great potential if it could be determined in a standard fashion and was inexpensive and easily measured.

Serum aspartate aminotransferase (AST) and alanine aminotransferase (ALT) are well-known liver enzymes released by both hepatocellular cells and nonhepatocellular cells, such as damaged myocardial cells, kidney, and skeletal muscle [2]. They are identified as indicators of various diseases, including liver disease, cardiovascular disease, cancer, and multiple organ dysfunction [3] and its association with prognosis [4]. The ratio of serum AST and ALT was initially described by De Ritis and has been known as the De Ritis ratio [5]. Over the past several decades, it has subsequently been shown to be a useful prognostic predictor in critical illness and malignant tumors, including acute myocardial infarction [6], cardiovascular surgery [7], metastatic renal cell carcinoma [8], and upper tract urothelial carcinoma [9].

Liver dysfunction is central in sHLH. Elevated liver enzymes, hepatomegaly, and coagulopathy are signs of liver involvement [10]. In fact, the inclusion of increased transaminase levels was supported by the 2009 ASH guidelines for the diagnosis of sHLH from Professor Filipovich [11]. Several observational studies indicated that patients with sHLH who had hepatic involvement had significantly worse overall survival than those patients without hepatic involvement [12, 13]. It is crucial for physicians to identify poor prognosis patients earlier after diagnosis and before treatment and to apply effective combination therapy. However, data identifying relevant hepatic prognostic factors for patients with sHLH are lacking. Thus, the aim of our study was to investigate the prognostic value of the De Ritis ratio in patients with sHLH.

## 2. Patients and Methods

### 2.1. Study Patients

**Figure 1:**
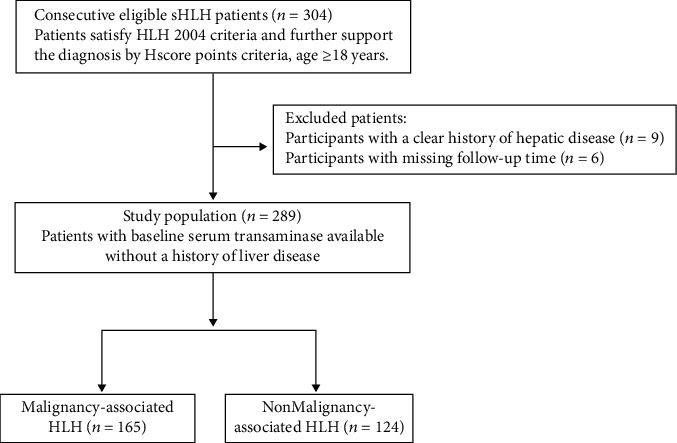
Consort diagram of enrolled patients. sHLH: secondary hemophagocytic lymphohistiocytosis.

This study was conducted by retrospectively reviewing clinical data from a total of 289 adult patients with sHLH who were diagnosed at the First Affiliated Hospital of Nanjing Medical University from September 2014 to December 2019. Our study was approved by the Ethics Committee of the First Affiliated Hospital of Nanjing Medical University (ChiCTR2000032421), and informed consent was obtained to review patient medical records. All eligible patients were assessed according to the Histiocytosis Society in 2004, and the Hscore criteria were further applied to support the diagnosis of sHLH. The exclusion criteria were as follows: (1) patients less than 18 years old, (2) patients without detailed serum aminotransaminase levels, (3) patients with a history of hepatitis or severe fatty liver, and (4) patients who refused any treatment. The detailed study flowchart is presented in Figure 1. This study was carried out in accordance with the Declaration of Helsinki and reported according to the Strengthening the Reporting of Observational studies in Epidemiology (STROBE) statement.

### 2.2. De Ritis Ratio

Laboratory data were routinely analyzed in each patient by standard clinical testing methodology in our hospital biochemical laboratory, and the upper limits of normal aminotransaminases were set at 40 and 50 IU/L for AST and ALT, respectively. AST/ALT (the De Ritis ratio) was categorized in tertiles according to the distributions of the study population: 1 st tertile ≤ 1.0, 1.0 < 2 st tertile ≤ 1.79, and 3 st tertile > 1.79.

### 2.3. Covariates

We included covariates that are major predictors of overall survival according to previous studies [14–16]. Detailed information on age, sex, complete blood cell counts, hepatosplenomegaly, and blood biochemical tests including triglycerides, lactate dehydrogenase (LDH), albumin (ALB), fibrinogen (FIB), ferritin, serum soluble interleukin-2 receptor (sIL-2R, sCD25), and *β*_2_-microglobulin (*β*_2_-MG) was reviewed through medical records. EBV was evaluated by both serology and EBV DNA real-time quantitative polymerase chain reaction (RQ-PCR) analysis. Bone marrow aspiration and biopsy samples were reviewed at the first diagnosis. Tumor or lymph node biopsy and PET/CT were identified as malignancy-associated hemophagocytic lymphohistiocytosis (MHLH) [17].

### 2.4. Endpoint and Follow-Up

The primary outcome was overall survival. The survival status of participants was confirmed with death records or a telephone call to the patients and patients' relatives, and overall survival was calculated as the time in days from the sHLH diagnosis to the date of death from any cause or the last follow-up.

### 2.5. Treatments

289 patients which include 165 MHLH and 124 non-MHLH. Among 165 patients, 128 (77.6%) had received systemic combination chemotherapy as initial therapy for sHLH, 33 patients were treated according to HLH-94 as initial therapy, and 4 patients with progressive multiple organ dysfunction who were treated with only high-dose immunoglobulin and glucocorticoid chemotherapy. In our 124 non-MHLH patients, 21 (16.9%) patients had HLH-94 therapy, 30 (24.2%) had received only glucocorticoid (GC) therapy combined high-dose immunoglobulin (IVIG), 24 (19.4%) had GC+IVIG +etoposide, 18 (14.5%) had GC+IVIG+etoposide+cyclosporine, 24 (19.4%) patients refused any remedies, and 7 (5.6%) patients only received anti-infective therapy. There were no differences in treatment regimens between three groups.

### 2.6. Statistical Analysis

Categorical variables are presented as frequencies and percentages. Comparisons across De Ritis ratio tertiles were performed using one-way ANOVA followed by the post hoc test (least significant difference) for continuous variables and chi-square statistics for categorical variables. Paired Spearman correlation coefficients between the De Ritis ratio and clinical characteristics were calculated. Kaplan-Meier curves for tertiles of the De Ritis ratio were plotted to display mortality rates. Adjusted survival curves are adjusted for covariates derived from the final model according to multivariable Cox proportional hazards models.

Univariate Cox regression analysis was used to estimate the relationship between potential predictors and survival status at long-term follow-up. Multivariable Cox regression with stepwise forward selection was performed to analyze the influence of relevant variables (variables with *P* < 0.05 in univariate Cox regression were subsequently entered into the model) on overall survival. The hazard ratio (HR) was estimated with 95% confidence intervals (CI), and the respective *P* values were reported from these analyses. HRs of ferritin and sCD25 variables refer to increases per 1000 *μ*g/L and 10,000 ng/L, respectively. We log_2_-transformed the De Ritis ratio because it was skewed. Restricted cubic splines with three knots placed at the 10^th^, 50^th^, and 90^th^ percentiles (the number of knots was selected according to the Akaike information criterion) were generated to examine the nonlinear relationships of the log_2_-transformed De Ritis ratio with the risk of mortality after adjusting for confounding factors, and the tests for nonlinearity were calculated by Wald *χ*^2^ tests. We regarded the De Ritis ratio tertiles as a continuous variable in the same model to test for significant linear trends. Moreover, we performed stratified analyses to explore whether the association between the De Ritis ratio and overall survival was modified by ALB, FIB, ferritin, hepatomegaly, EBV, etiologies, and treatment intervention. Interaction analyses were tested between De Ritis ratio tertiles and all other covariates in stratified analyses.

All statistical analyses were performed with *R* software version 3.6.0 (R Foundation for Statistical Computing, Vienna, Austria) and STATA/MP statistical software (version 14.0; stataCorp, TX, USA), and two-sided *P* values <0.05 were considered statistically significant.

## 3. Results

### 3.1. Distribution of the De Ritis Ratio and Patient Characteristics

**Figure 2:**
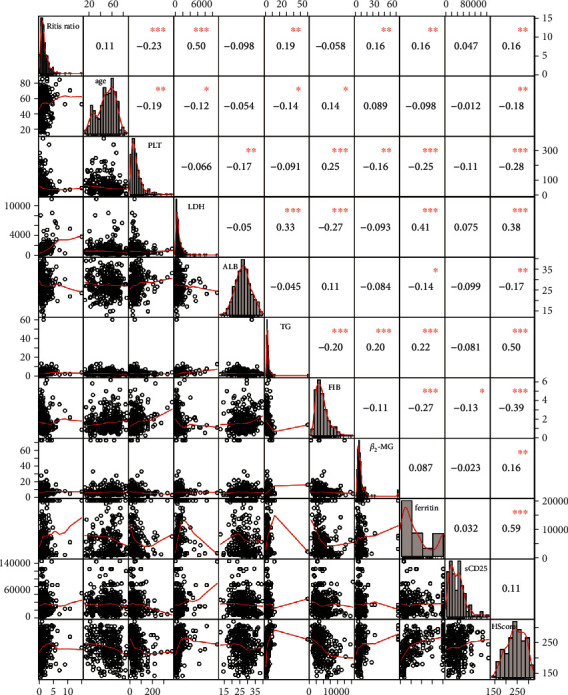
Pairwise correlation between the De Ritis ratio (AST/ALT) and age, platelet (PLT), lactate dehydrogenase (LDH), albumin (ALB), triglyceride (TG), fibrinogen (FIB), beta2-microglobulin (*β*_2_-MG), ferritin, Soluble IL-2 receptor (sCD25), and Hscore points.

Detailed baseline characteristics of 289 study participants stratified by tertiles of the De Ritis ratio are shown in Table 1. The median age of the participants was 53 (interquartile range, 41–64 years), and 61.9% were male. For the study population, the median De Ritis ratio level was 1.34 (0.84-2.29). Participants with higher levels of the De Ritis ratio had higher LDH, TG, ferritin, *β*_2_-MG, and Hscore points and a higher proportion of MHLH. In addition, PLT was significantly lower in patients with the 3 st tertile of the De Ritis ratio (*P* < 0.001). Interestingly, we observed that the 1 st tertile of the De Ritis ratio individuals had a higher prevalence of hemophagocytes (Table 1). Additionally, the De Ritis ratio showed a strong positive correlation with LDH values (*r* = 0.502; *P* < 0.001), TG values (*r* = 0.190; *P* = 0.001), *β*_2_-MG (*r* = 0.159; *P* = 0.007), ferritin (*r* = 0.163; *P* = 0.005), and Hscore points (*r* = 0.160; *P* = 0.007). The De Ritis ratio was inversely associated with PLT (*r* = 0.232; *P* < 0.001) (Figure 2).

### 3.2. Associations of the De Ritis Ratio with Overall Survival

**Figure 3:**
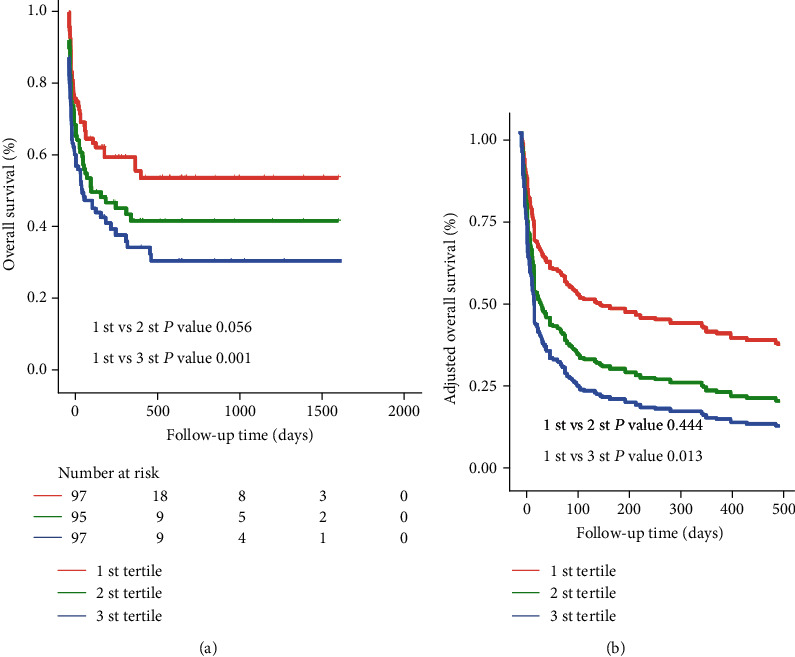
Survival curves in all sHLH patients according to De Ritis ratio tertiles. (a) Kaplan-Meier survival curves. (b) Adjusted survival curves: curves were adjusted for hepatomegaly, albumin (ALB), fibrinogen (FIB), EBV, ferritin, malignancy-associated HLH (MHLH), and treatment strategies.

**Figure 4:**
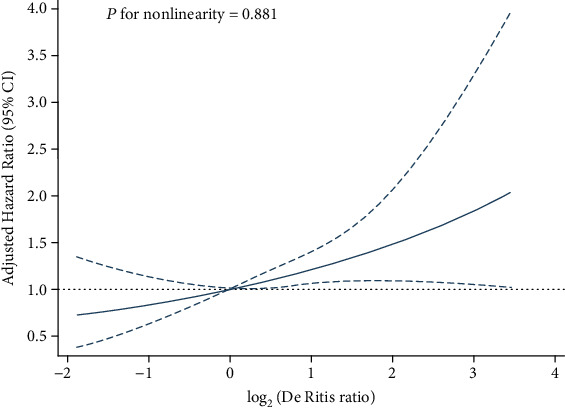
Cubic spline plot of the association between the log_2_-transformed De Ritis ratio and the risk of mortality among adult onset HLH. The solid line and dashed line represent the estimated hazard ratios and their corresponding 95% CIs. Analyses were adjusted for hepatomegaly, albumin (ALB), fibrinogen (FIB), EBV, ferritin, malignancy-associated HLH (MHLH), and treatment strategies.

**Figure 5:**
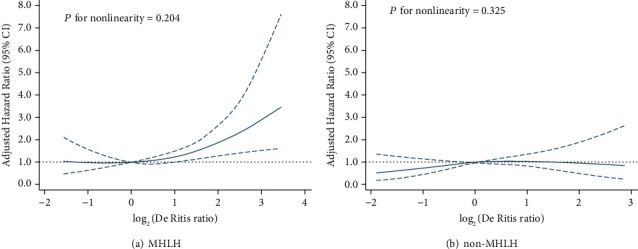
Cubic spline plots of the association between the log_2_-transformed De Ritis ratio and the risk of mortality among adult onset MHLH (a) and non-MHLH (b). Analyses were adjusted for hepatomegaly, albumin (ALB), fibrinogen (FIB), EBV, ferritin, malignancy-associated HLH (MHLH), and treatment strategies.

After a median follow-up of 60 days (range 17-227.5), we observed 205 deaths (134 for MHLH and 71 for non-MHLH). The Kaplan–Meier survival curves for tertiles of the De Ritis ratio in patients were plotted (Figure 3). Patients in the 1 st and 2 st tertiles had a similar risk of death (*P* = 0.056), while patients in the 3 st tertile had a significantly greater risk of death (HR = 1.8, 95% CI: 1.3–2.5, *P* = 0.001) compared with the 1 st tertile; after further adjusting for confounding factors, the HRs with 95% CIs of mortality for the 2 st and 3 st tertiles were 1.2 (0.8-1.7) and 1.6 (1.1–2.2), respectively (*P* for trend <0.01) (Table 2 and Figure 3). RCS plots showed a positive linear association between the log2-transformed De Ritis ratio and the risk of mortality in sHLH (Figure 4, *P* for nonlinearity =0.881). Meanwhile, a similar trend was also found in the MHLH group rather than the non-MHLH group when we further considered etiologies (Figure 5).

### 3.3. Subgroup Analysis

**Figure 6:**
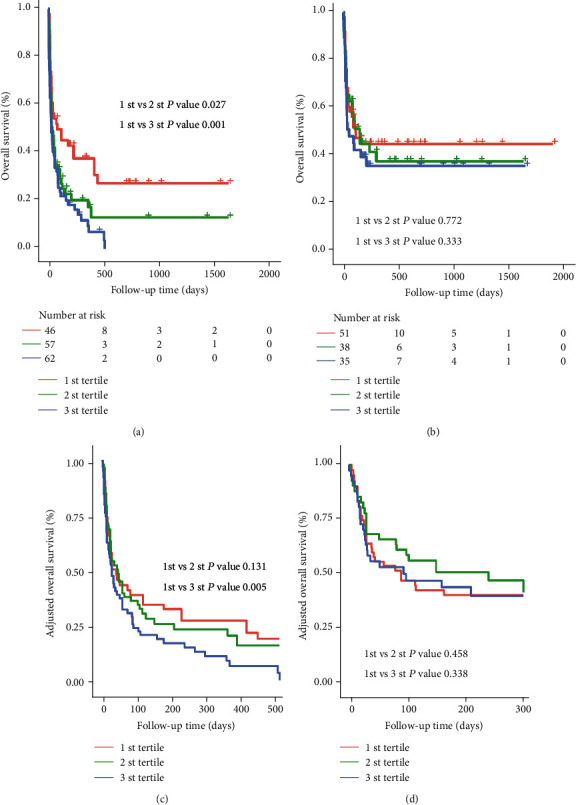
Survival curves in patients with HLH according to De Ritis ratio tertiles. (a, b) Kaplan-Meier survival curves in MHLH and non-MHLH. (c, d) Adjusted survival curves in MHLH and non-MHLH: curves are adjusted for hepatomegaly, albumin (ALB), fibrinogen (FIB), EBV, ferritin, malignancy-associated HLH (MHLH), and treatment strategies.

We performed stratified analyses between the De Ritis ratio and the risk of mortality by ALB, FIB, ferritin, hepatomegaly, EBV, etiologies, and treatment intervention. In the subgroup analyses, patients with ferritin > 10,000 *μ*g/L, sCD25 ≤ 20,000 ng/L, those without EBV infection, MHLH, and patients who received therapeutic intervention had similar worse survival between the 1 st tertile and 2 st tertile, while patients in the 3 st tertile of the De Ritis ratio had a significantly higher risk of death compared with the 1 st tertile (Table 3). Although the interaction of the De Ritis ratio with etiologies was not significant, aetiology-stratified models suggested that the association of the De Ritis ratio and mortality was significant among patients with MHLH but not non-MHLH patients (Figure 6).

## 4. Discussion

To the best of our knowledge, this is the first study to assess the impact of baseline aspartate transaminase/alanine transaminase (AST/ALT, De Ritis ratio) on sHLH patient outcomes before treatment. The De Ritis ratio has a strong impact on mortality, persisting regardless of adjusting treatment, etiologies, EBV, ferritin, albumin, fibrinogen, and hepatomegaly. Moreover, by restricted cubic spline (RCS) modeling, we also found a direct association between the De Ritis ratio and mortality in the total cohort, similar to the MHLH and non-MHLH subgroups. Multivariable regression analysis and adjusted Kaplan-Meier curves also revealed a higher risk of mortality in patients with the highest De Ritis ratio.

The De Ritis ratio provided incremental values for risk prediction beyond clinical risk factors. These results are consistent with prior findings of the De Ritis ratio and a high mortality rate in critical illness and various types of cancers [6, 7, 18, 19]. While in hematologic malignancies, the De Ritis ratio was reported only as an adverse event style after treatment [20, 21], such as leukaemia and lymphoma, and its association with mortality has not been researched. In our study, we found that an elevated De Ritis ratio has a significantly positive correlation with inferior overall survival. Compared with the 1 st tertile, the 2 st tertile and 3 st tertile were associated with a 20% and 60% increase in the risk of mortality, respectively. The Max study and the Lee study also showed similar findings in critically ill patients with cardiovascular disease [6, 7]. Furthermore, we also found a new prospective relationship between the De Ritis ratio and different clinical parameters for sHLH, which has a positive association with higher TG, increased ferritin, elevated LDH and even Hscore points, and an inverse association with decreased PLT. Moreover, unadjusted and adjusted survival analysis also demonstrated that a higher De Ritis ratio predicted poor overall survival, the same as in the MHLH and non-MHLH subgroups. The present study was able to illustrate that the De Ritis ratio is a prognostic indicator.

The underlying pathophysiologic mechanism of elevated transaminase and its prognostic value in patients with sHLH is still unclear. In patients with sHLH, activated lymphocytes and macrophages secrete high levels of pro- and anti-inflammatory cytokines and chemokines, leading to liver involvement and progressive multiple organ dysfunction syndrome [10, 22]. One possible speculation is that periportal lymphocytic infiltration could aberrantly damage liver cells and promote further mitochondrial dysfunction, resulting in the accumulation of serum transaminase [23, 24]. In addition, IFN-*γ* has been proven to be a key cytokine that drives disease development in sHLH [25], stimulates hepatic inflammation by increasing TNF and IL-6, and induces hepatocyte apoptosis via a BCL2-mediated pathway [26]. Moreover, an elevated level of AST compared with ALT is hypothesized to be associated with increased cancer cell high proliferative status and normal tissue damage and can result in the higher activation of circulating AST [4], in line with a large part of the distribution in our 165 patients with MHLH. Finally, elevated AST levels may indicate a hypoperfusion state due to the cytokine storm leading to multiorgan dysfunction syndrome because AST is widely expressed in various types of tissues, whereas ALT is enriched in liver tissues.

Studies focused on liver involvement, including hepatomegaly and elevated liver enzymes, have been reported to be associated with poor outcome in HLH [10, 16, 27]. Nevertheless, although the single measures of AST or ALT are associated with prognosis, they are susceptible to various related factors, such as alcoholic liver disease and skeletal muscle-related disease [28]. Calculating the AST/ALT ratio reflects the combination of AST and ALT and is a stable factor [29], thus making the De Ritis ratio to be a promising biomarker for HLH.

Our findings provide references that routine clinical laboratory assays such as liver function tests can be used to identify patients at higher risk of death while applying an advanced evaluation for sHLH. On the one hand, in our study, an De Ritis ratio in the 3 st tertile had the worst overall survival outcome. This leads us to the hypothesize that the De Ritis ratio might be a particularly valuable tool in identifying the highest risk patients with sHLH. Moreover, 3 st tertile of the De Ritis ratio had lower platelet levels, higher TG, increased ferritin, and elevated LDH in terms of laboratory findings, which are related to disease activation and hyper-inflammatory status that have also been observed as being associated with a worse prognosis in other studies [10, 15]. In these patients, it may provide clinical guidance that timely multidisciplinary discussion of initiating HLH-directed immune suppressants and more support treatment is needed prior to the finalized results of specialized assays. In addition, the use of the De Ritis ratio also has the advantage of being an easily assessable biomarker at a low cost. However, there are several limitations in our study. First, this is a retrospective and single-centre study, and the results are subject to potential bias. Second, although a broad set of covariates was included in regression models, unmeasured confounding by additional factors could also play a role.

In conclusion, the De Ritis ratio is an independent prognostic factor in patients with sHLH. Further studies are warranted.

## Figures and Tables

**Table 1:** Baseline demographic, clinical, and laboratory characteristics of the study patients according to tertiles of AST/ALT (De-Ritis ratio).

	1 st tertile (*N* = 97)	2 st tertile (*N* = 95)	3 st tertile (*N* = 97)	*P* value
De Ritis ratio	0.68 (0.50-0.85)	1.34 (1.17-1.58)	2.89 (2.28-3.96)	<0.001
ALT, U/L	90.9 (52.0-193.2)	67.9 (37.3-147.9)	46.2 (26.9-98.4)	<0.001
AST, U/L	55.9 (32.2-125.9)	96.4 (49.6-196.6)	140.0 (81.40-288.0)	<0.001
Male, *n* (%)	58 (59.8)	65 (68.4)	56 (57.7)	0.271
Age, years	49 (37-64)	55 (43-66)	58 (42-65)	0.074
ANC, ×10^9^/L	1.13 (0.82-2.73)	0.98 (0.69-1.92)	0.89 (0.69-2.32)	0.132
HB, g/L	89 (74-103)	85 (69-104)	83 (70-94)	0.277
PLT, ×10^9^/L	61 (35-90)	41 (24-61)	36 (20-60)	<0.001
LDH, U/L	452 (336-660)	676 (382-1025)	1195 (683-1990)	<0.001
ALB, g/L	27.60 (5.01)	27.02 (5.47)	26.87 (4.59)	0.568
TG, mmol/L	2.36 (1.60-3.57)	2.35 (1.63-3.27)	2.86 (2.00-4.43)	0.002
FIB, g/L	1.66 (1.11-2.32)	1.44 (0.91-2.12)	1.46 (1.05-2.18)	0.466
Ferritin, ug/L	3137 (1321-8024)	5043 (2193-14576)	6258 (1600-17400)	0.016
sCD25, ng/L	31212 (17540-43915)	34687 (16915-51866)	37622 (17744-54064)	0.607
Splenomegaly (%)	85 (87.6)	82 (86.3)	89 (91.8)	0.465
Hepatomegaly (%)	15 (15.5)	21 (22.1)	16 (16.5)	0.437
Lymphadenopathy (%)	42 (43.3)	56 (58.9)	53 (54.6)	0.08
Hemophagocytic (%)	91 (93.8)	86 (90.5)	80 (82.5)	0.035
HScore, points	224 (196-258)	239 (207-269)	244 (220-274)	0.011
EBV infection (%)	45 (46.4)	56 (58.9)	45 (46.4)	0.134
Etiology				0.053
MHLH (%)	46 (47.4)	57 (60.0)	62 (63.9)	
Non-MHLH (%)	51 (52.6)	38 (40.0)	35 (36.1)	
Chemotherapy (%)	86 (88.7)	88 (92.6)	84 (86.6)	0.39

Abbreviations: ALT: alanine transaminase; AST: aspartate transaminase; De-Ritis ratio: aspartate transaminase/alanine transaminase; ANC: absolute neutrophil count; HB: hemoglobin; PLT: platelet; LDH: lactate dehydrogenase; ALB: albumin; TG: triglyceride; FIB: fibrinogen; sCD25: soluble interleukin-2 receptor; *β*_2_-MG: beta_2_-microglobulin; EBV: Epstein-Barr virus; MHLH: malignancy-associated hemophagocytic lymphohistiocytosis; Non-MHLH: nonmalignancy associated hemophagocytic lymphohistiocytosis.

**Table 2:** Uni- and multivariate Cox regression analyses of overall survival.

	Unadjusted		Adjusted	
	HR (95% CI)	*P*	HR (95% CI)	*P*
Male	1.5 (1.1-2.0)	0.011		
Age > 60 years	1.3 (0.9-1.7)	0.065		
ANC > 1.0 × 109/L	1.2 (0.9-1.5)	0.294		
HB < 90 g/L	1.4 (1.0-1.8)	0.030		
PLT < 100 × 10^9^/L	1.9 (1.2-3.0)	0.004		
FIB ≤ 1.5 g/L	1.9 (1.4-2.5)	<0.001	1.4 (1.0-1.9)	0.031
LDH ≥ 2.5 × ULN	1.1 (0.9-1.5]	0.390		
ALB < 25 g/L	1.7 (1.3-2.2)	<0.001	1.5 (1.1-2.0)	0.008
TG ≥ 3.0 mmol/L	1.2(0.9-1.6)	0.244		
Splenomegaly	1.1 (0.7-1.7)	0.651		
Hepatomegaly	1.8 (1.3-2.5)	0.001	1.6 (1.1-2.2)	0.011
Lymphadenopathy	1.2 (0.9-1.5)	0.268		
Hemophagocytosis	0.9 (0.6-1.3)	0.436		
Ferritin > 10000ug/L	1.9 (1.4-2.6)	<0.001	1.9 (1.4-2.6)	<0.001
sCD25 > 20000 ng/L	1.6 (1.1-2.1)	*0.005*		
EBV infection	1.8 (1.4-2.4)	<0.001	1.5 (1.1-2.0)	0.009
MHLH	1.8 (1.3-2.4)	<0.001	1.8 (1.3-2.5)	<0.001
Chemotherapy	0.9 (0.6-1.4)	0.670	0.4 (0.3-0.7)	<0.001
De-Ritis ratio				
≤1.00	1 (ref)		1 (ref)	
1.01 < ratio ≤1.79	1.4 (1.0-2.0)	0.056	1.2 (0.8-1.7)	0.444
>1.80	1.8 (1.3-2.5)	0.001	1.6 (1.1-2.2)	0.013

Abbreviations: ANC: absolute neutrophil count; HB: hemoglobin; PLT: platelet; LDH: lactate dehydrogenase; ALB: albumin; TG: triglyceride; FIB: fibrinogen; sCD25: soluble interleukin-2 receptor; EBV: Epstein-Barr virus; MHLH: malignancy-associated hemophagocytic lymphohistiocytosis; De-Ritis ratio: aspartate transaminase/alanine transaminase; HR: hazards ratio; 95% CI: 95% confidence interval.

**Table 3:** Stratified associations between De Ritis ratio and overall survival.

Subgroups	1 st tertile	2 st tertile	3 st tertile	*P* for trend	*P* for interaction
	OR (95% CI)	OR (95% CI)	OR (95% CI)		
Gender					0.874
Male	1 (ref)	0.8 (0.5-1.3)	1.4 (0.9-2.1)	0.175	
Female	1 (ref)	1.2 (0.6-2.5)	1.4 (0.7-2.8)	0.317	
Age ≤ 60 years					0.950
Yes	1 (ref)	1.1 (0.7-1.8)	1.4 (0.9-2.2)	0.203	
No	1 (ref)	1.0 (0.5-2.0)	1.5 (0.8-2.7)	0.168	
PLT ≤ 100 × 10^9^/L					0.095
Yes	1 (ref)	1.0 (0.7-1.5)	1.3 (0.9-1.9)	0.157	
No	1 (ref)	0.4 (0.07-2.6)	3.2 (0.6-16.9)	0.212	
Ferritin > 10000ug/L					0.985
Yes	1 (ref)	1.2 (1.0-4.6)	2.1 (1.0-4.6)	0.035	
No	1 (ref)	1.0 (0.7-1.6)	1.4 (0.9-2.1)	0.165	
sCD25 > 20000 ng/L					0.249
Yes	1 (ref)	0.8 (0.5-1.3)	1.1 (0.7-1.7)	0.470	
No	1 (ref)	1.6 (0.8-3.3)	2.6 (1.3-5.2)	0.009	
EBV infection					0.329
Yes	1 (ref)	1.1 (0.7-1.8)	1.1 (0.7-1.8)	0.634	
No	1 (ref)	1.1 (0.6-2.0)	2.1 (1.2-3.6)	0.005	
MHLH					0.209
Yes	1 (ref)	1.2 (0.7-1.9)	1.8 (1.1-2.8)	0.014	
No	1 (ref)	0.8 (0.4-1.5)	1.1 (0.6-1.9)	0.496	
Chemotherapy					0.748
Yes	1 (ref)	1.2 (0.8-1.8)	1.5 (1.1-2.2)	0.024	
No	1 (ref)	0.04 (0.003-0.5)	1.4 (0.4-5.7)	0.188	

Analyses were adjusted for hepatomegaly, albumin (ALB), fibrinogen (FIB), EBV, ferritin, malignancy-associated HLH (MHLH), and treatment strategies when they were not stratified variables.

## Data Availability

To request access to the data, please contact the corresponding author, who is the principal investigator of the study and responsible for the consortium dataset.
